# Association Between Eating Disorders and Subsequently Recorded Depression and Bipolar Disorder: A Retrospective Cohort Study

**DOI:** 10.3390/nu18203404

**Published:** 2026-10-17

**Authors:** Vanessza Hadar, Battamir Ulambayar, Attila Csaba Nagy, Amr Sayed Ghanem

**Affiliations:** 1 Department of Dietetics, Institute of Health Sciences, Faculty of Health Sciences, University of Debrecen, Kassai Way 26, 4028 Debrecen, Hungary; hadar.vanessza@etk.unideb.hu; 2 Department of Epidemiology, Institute of Health Sciences, Faculty of Health Sciences, University of Debrecen, Kassai Way 26, 4028 Debrecen, Hungary; ulambayar.battamir@etk.unideb.hu (B.U.); nagy.attila@etk.unideb.hu (A.C.N.)

**Keywords:** eating disorder, mood disorder, depression, bipolar disorder, retrospective cohort, survival analysis, exposure

## Abstract

Background/Objectives: Mental health is strongly affected by eating disorders, yet there are few longitudinal studies that have examined their association with subsequently recorded mood disorders. The present study aimed to investigate the association between exposure to eating disorders and first recorded diagnoses of depression and bipolar disorder during follow-up. Methods: This retrospective cohort study was based on the hospital database of the Clinical Center of the University of Debrecen, covering the period between 2007 and 2022. The dataset included 333,807 patients and 805,989 hospital records. Diagnoses were identified using physician-recorded ICD-10 codes. Eating disorders were defined as the exposure variable, while first recorded diagnoses of depression and bipolar disorder during follow-up were treated as outcomes. Patients with the relevant mood disorder recorded in their entry year were excluded from the corresponding analysis. Associations were evaluated using survival analysis to estimate hazard ratios (aHRs) and 95% confidence intervals (CIs). Results: In the respective Cox analysis samples, eight subsequent depression diagnoses occurred among 68 patients with baseline eating disorders, and seven subsequent bipolar disorder diagnoses occurred among 82 patients with baseline eating disorders. Eating disorders were associated with higher hazards of subsequently recorded depression (aHR = 4.23; 95% CI: 2.11–8.46) and bipolar disorder (aHR = 5.45; 95% CI: 2.59–11.44). Female sex was associated with a higher hazard of subsequently recorded depression and a lower hazard of subsequently recorded bipolar disorder. Conclusions: Recorded eating disorders were associated with subsequently recorded depression and bipolar disorder. These associations require cautious interpretation given the small number of exposed outcome events, imprecise estimates and potential residual confounding, and warrant further prospective investigation.

## 1. Introduction

Mental disorders are among the leading causes of disability worldwide, and the burden has increased over recent decades despite relatively stable age-standardized rates [1]. One such condition is major depressive disorder, which is the single largest contributor to years lived with disability and affects more than 322 million people worldwide [2]. Bipolar disorder, less common with an estimated lifetime prevalence of 1–2%, is associated with recurrent episodes, significant functional impairment and one of the highest suicide risks among psychiatric illnesses [3].

Eating disorders such as anorexia nervosa, bulimia nervosa and binge-eating disorder are less prevalent, but are some of the most severe psychiatric disorders due to the high morbidity, mortality and long-term psychosocial consequences [4,5,6]. Beyond disturbances in eating behavior, eating disorders are associated with impaired quality of life, increased healthcare utilization, and substantial psychiatric comorbidity [7]. Delayed recognition and help-seeking are common in eating disorders, with stigma, shame, limited recognition of illness severity, and sociocultural influences contributing to barriers to seeking professional help [8].

Psychiatric comorbidity is central to the clinical burden of eating disorders. In a nationally representative study of US adults, anorexia nervosa, bulimia nervosa, and binge-eating disorder were each associated with lifetime mood disorders, with major depressive disorder being the most prevalent psychiatric comorbidity across these eating disorder groups [9]. In a clinical cohort of 2929 patients with bipolar disorder, Flaudias et al. identified lifetime eating disorders in 16.4% of participants, with a higher prevalence in bipolar II than bipolar I disorder [10]. These findings demonstrate substantial clinical overlap, although comorbidity estimates alone do not establish the temporal sequence of the disorders.

Longitudinal evidence also suggests a bidirectional relationship. A systematic review and meta-analysis by Puccio et al. found that eating pathology predicted subsequent depression and that depression predicted subsequent eating pathology [11]. Accordingly, the association may reflect reciprocal influences, shared vulnerability, or both [11,12]. An important remaining question is whether a recorded eating disorder diagnosis is associated with subsequent first-recorded depression or bipolar disorder in routine healthcare data. Examining this sequence can extend existing comorbidity research, although the temporal ordering of recorded diagnoses does not necessarily establish the order of clinical onset or a causal relationship.

Large electronic healthcare databases provide an opportunity to address this knowledge gap by enabling longitudinal follow-up of large patient populations under routine clinical conditions. Because physician-recorded diagnoses are documented over extended periods, these databases allow temporal sequencing between exposure and outcome while reflecting real-world healthcare practice. Such data can therefore complement findings from traditional cohort studies and improve understanding of longitudinal associations between recorded eating disorders and mood disorder diagnoses [13].

The primary objective of the present study was to examine the association between recorded eating disorders at baseline and first recorded diagnoses of depression and bipolar disorder during follow-up in a retrospective cohort derived from routine hospital care. We hypothesized that patients with a recorded baseline eating disorder would have higher hazards of these outcomes than patients without a recorded baseline eating disorder.

## 2. Materials and Methods

### 2.1. Study Population and Data Source

This retrospective longitudinal study was conducted using electronic hospital records from the Clinical Center of the University of Debrecen, Hungary, covering the period between 2007 and 2022. The database contained information on 333,807 unique patients and 805,989 hospital records documenting diagnostic and clinical events. After data cleaning, 7490 first recorded depression diagnoses and 3552 first recorded bipolar disorder diagnoses were identified during follow-up in the respective analytic populations. Baseline ED diagnoses were recorded for 140 patients in the initial depression cohort and 172 patients in the initial bipolar disorder cohort. Diagnoses were identified using physician-recorded ICD-10 codes in the hospital information system. The investigators accessed a secondary dataset containing ICD-10-coded diagnoses and associated demographic and temporal information, without access to individual medical charts. BMI and medication-related data were unavailable, and detailed nutritional and metabolic measurements could not be obtained from the accessible dataset.

The completed STROBE checklist for cohort studies is provided in Supplementary Table S1.

### 2.2. Exposure and Outcome Definitions

Eating disorders were defined using ICD-10 codes F50.0, F50.1, F50.2, F50.3, F50.4, F50.5, F50.8, and F50.9. ED exposure was identified from recorded diagnostic codes. Accordingly, the comparison group comprised patients without a recorded qualifying ED diagnosis, rather than patients confirmed to be free of eating disorders. Medical charts and standardized screening information were unavailable for independent validation of exposure classification.

The outcomes were first recorded diagnoses of depression or bipolar disorder during follow-up. Depression was defined using ICD-10 codes F32 and F33, while bipolar disorder was defined using code F31. Patients with the relevant mood disorder recorded in their first observed calendar year were excluded from the corresponding analysis, and outcomes were first recorded diagnoses in subsequent calendar years. No fixed pre-entry washout period was required. Patients were included in the Cox analyses if their recorded observation period spanned at least two different calendar years, corresponding to positive observed follow-up, and complete data for the model covariates were available. The index year was each patient’s first recorded calendar year in the dataset, using the same definition for both exposure groups. ED status was determined from qualifying diagnoses recorded in that year and treated as a fixed baseline exposure. Patients without an entry-year ED diagnosis formed the comparison group; ED diagnoses first recorded during later follow-up did not change their baseline classification. Follow-up was measured in calendar-year units from the index year until the first recorded outcome or last available observation. Vital status and dates of death were unavailable in the secondary dataset; therefore, deaths could not be identified or incorporated into a competing-risk analysis.

### 2.3. Covariates

Demographic covariates included sex and age, while hypertension status was included as a clinical covariate. Age was entered into the models categorically (0–17, 18–44, 45–64 and ≥65 years), with 0–17 years as the reference category. These cut-offs provided pragmatic groupings for comparisons across childhood/adolescence, younger adulthood, middle adulthood and older adulthood. Sex and hypertension were coded as binary variables.

Hypertension was included as a clinical covariate and a limited indicator of recorded somatic disease burden. Direct measures of healthcare utilization, such as the number or frequency of healthcare contacts, were unavailable.

### 2.4. Statistical Analysis

All analyses were performed using Stata version 18 [14]. Time-to-event analyses were conducted to examine the association between eating disorders and subsequent mood disorders.

Multivariable associations were assessed using Cox proportional hazards regression models, with results reported as hazard ratios (HRs) and 95% confidence intervals (CIs). The proportional hazards assumption was assessed using Schoenfeld residuals for the ED exposure and the other covariates. Participant counts, outcome events, person-years of observation, and crude rates of first recorded diagnoses per 1000 person-years with 95% confidence intervals were summarized by baseline ED status within each Cox estimation sample.

**Figure 1:**
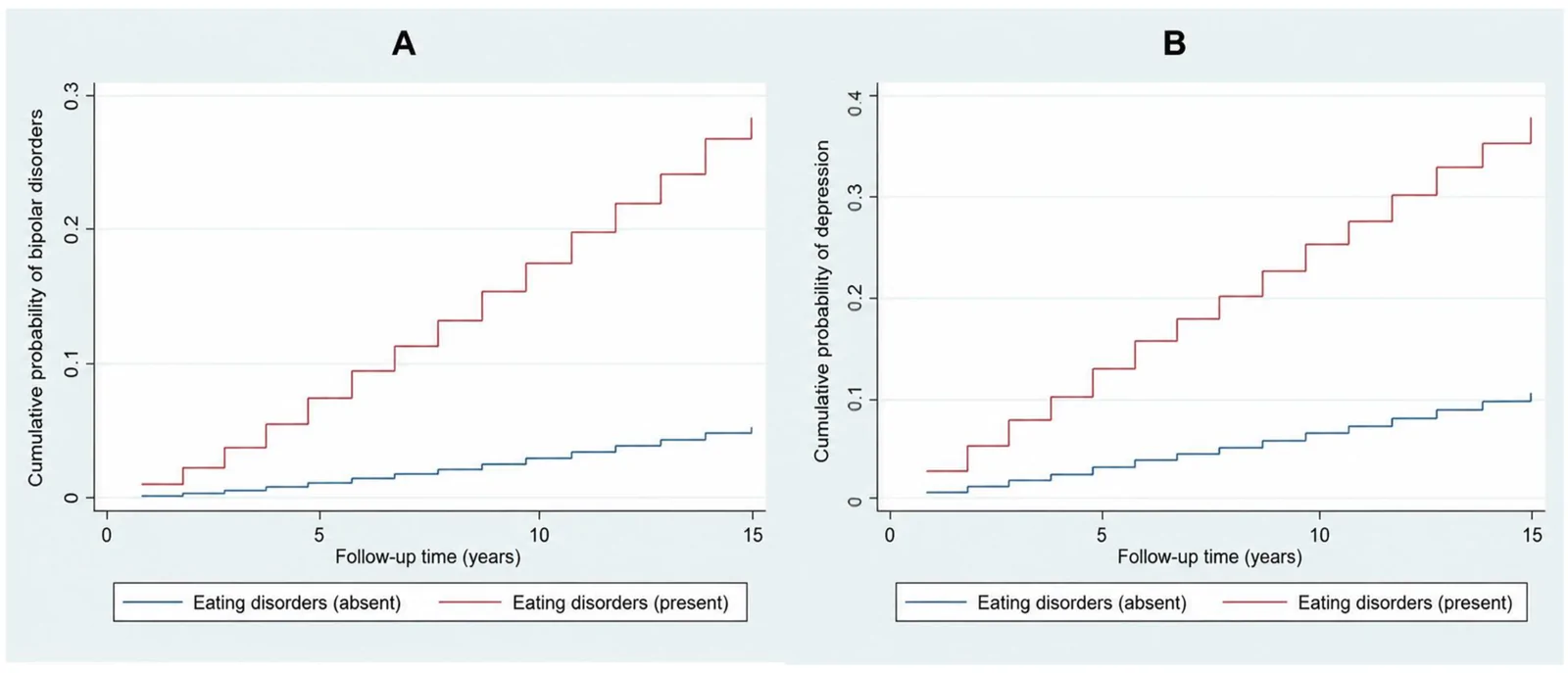
Model-based failure probabilities, calculated as 1 − S(t), for a recorded diagnosis of (**A**) bipolar disorder and (**B**) depression, with eating disorder status set to absent or present.

**Figure 2:**
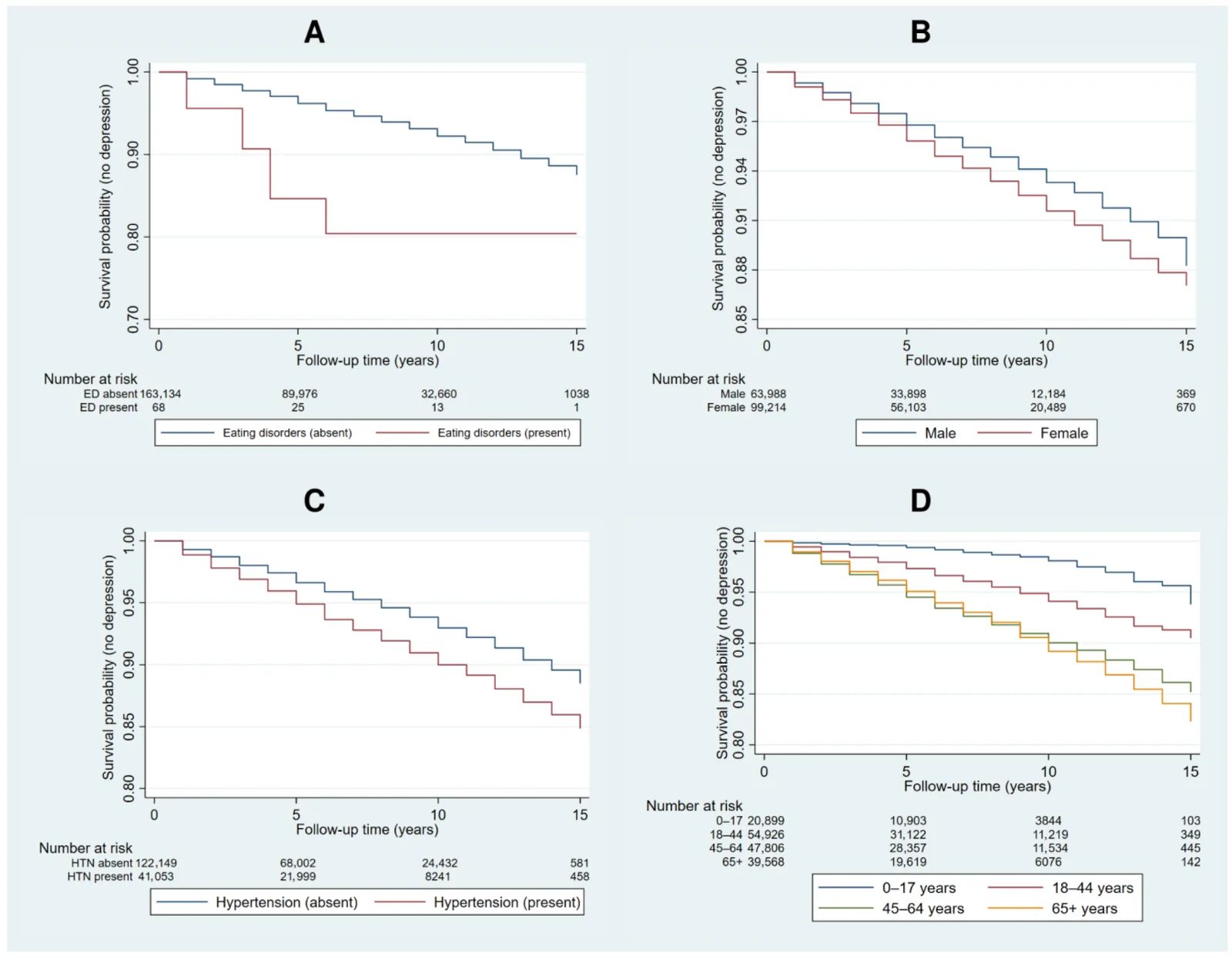
Kaplan–Meier estimates of remaining free from a recorded depression diagnosis, stratified by (**A**) eating disorder status, (**B**) sex, (**C**) hypertension status, and (**D**) age group. Numbers at risk at 0, 5, 10 and 15 years are shown below each panel. *Y*-axis ranges differ between panels.

**Figure 3:**
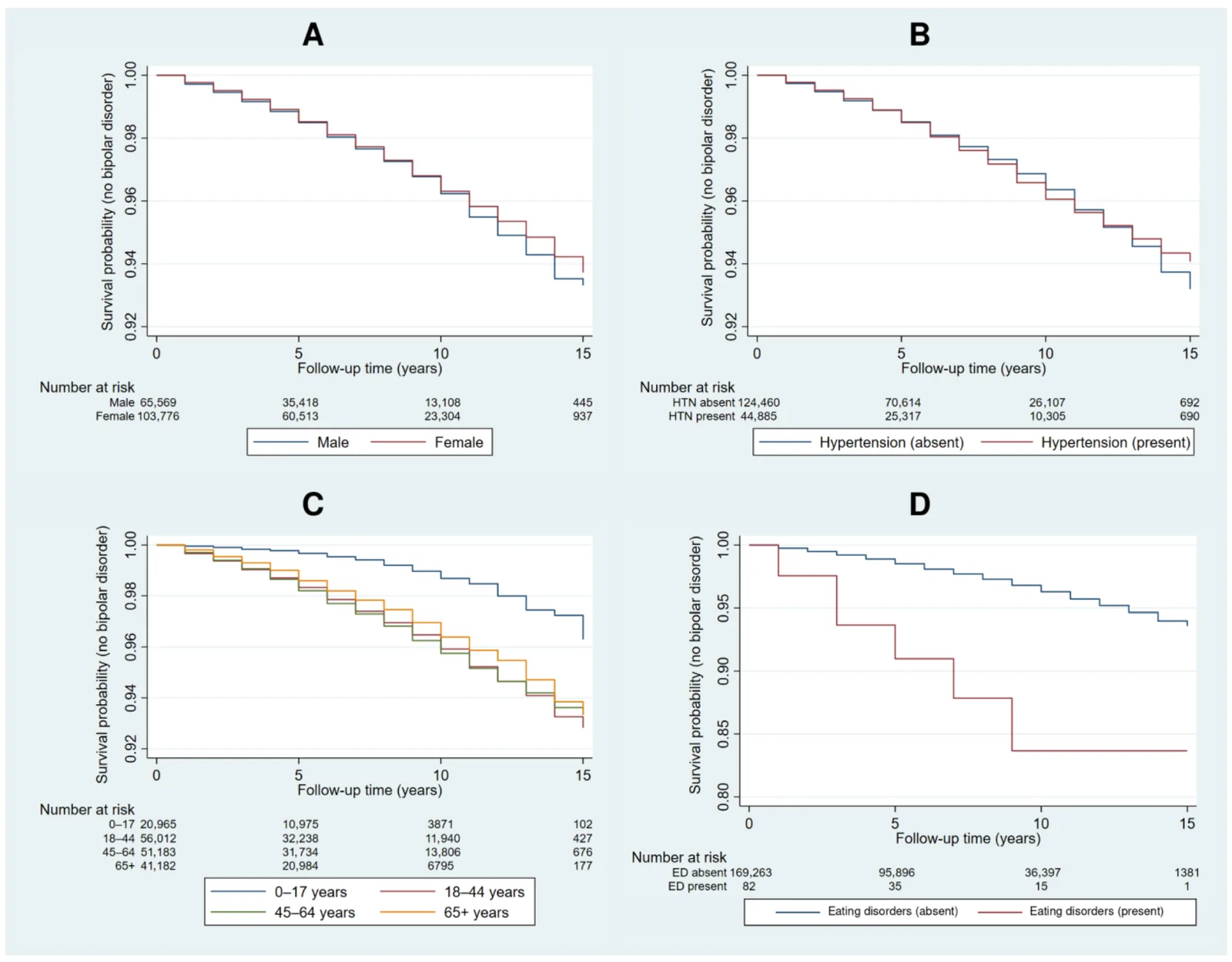
Kaplan–Meier estimates of remaining free from a recorded bipolar disorder diagnosis, stratified by (**A**) sex, (**B**) hypertension status, (**C**) age group, and (**D**) eating disorder status. Numbers at risk at 0, 5, 10 and 15 years are shown below each panel. *Y*-axis ranges differ between panels.

Model-based failure probabilities, 1 − S(t), were plotted by eating disorder status (Figure 1). These failure probabilities were not competing-risk cumulative incidence estimates. Kaplan–Meier curves were used to estimate the probability of remaining free from a recorded outcome diagnosis across exposure groups and covariate strata, with numbers at risk shown at 0, 5, 10 and 15 years (Figure 2 and Figure 3).

All statistical tests were two-sided, and *p* < 0.05 was considered statistically significant.

Exploratory Cox regression analyses were attempted within age groups and eating disorder subtypes to investigate potential heterogeneity in the associations. However, these models did not converge in the setting of sparse ED-exposed observations and outcome events within the resulting subgroups. Estimates from non-convergent models were not interpreted or reported.

## 3. Results

### 3.1. Baseline Characteristics of the Study Population

The study population (Table 1) included 323,120 individuals in the depression-related cohort and 331,062 individuals in the bipolar disorder-related cohort. In both cohorts, the largest age group was 18–44 years, representing 34.67% (*n* = 112,033) of the depression cohort and 34.25% (*n* = 113,378) of the bipolar disorder cohort. Females represented a slightly higher proportion of both cohorts, accounting for 56.49% (*n* = 182,546) and 56.9% (*n* = 188,358), respectively. A qualifying eating disorder diagnosis was recorded for 140 patients (0.04%) in the depression cohort and 172 patients (0.05%) in the bipolar disorder cohort. These percentages describe the proportion identified through diagnostic coding in the respective cohorts and should not be interpreted as estimates of the underlying prevalence of eating disorders. Overall, the two cohorts showed highly similar demographic and clinical distributions. After restricting to patients with positive follow-up and complete model covariates, the final Cox analyses included 163,199 patients for depression and 169,342 for bipolar disorder. These samples included 68 patients with baseline ED and eight subsequent depression diagnoses, and 82 patients with baseline ED and seven subsequent bipolar disorder diagnoses. Event counts, person-time, and crude rates by ED status are presented in Supplementary Table S2. Baseline ED code-specific patient counts and subsequent recorded mood disorder events within the final Cox analysis samples are presented in Supplementary Table S3.

Participant selection is summarized in Supplementary Figure S1. In the depression and bipolar disorder cohorts, respectively, 159,918 and 161,717 patients were excluded because they had no positive observed follow-up; three additional patients in each cohort were excluded because of incomplete model covariates. Compared with excluded patients, those included in the Cox analyses were more often aged ≥45 years (depression: 53.54% versus 45.61%; bipolar disorder: 54.54% versus 46.00%), female (60.79% versus 52.11%; 61.28% versus 52.30%, respectively), and hypertensive (25.16% versus 20.04%; 26.51% versus 20.41%, respectively). Recorded ED frequencies were below 0.1% in both included and excluded groups.

### 3.2. Model-Based Failure Probabilities by Eating Disorder Status

Figure 1 presents model-based failure probabilities, 1 − S(t), for a recorded diagnosis of bipolar disorder (Panel A) and depression (Panel B), with eating disorder status set to absent or present. For both outcomes, predicted failure probabilities were higher with eating disorder status set to present, with separation increasing over follow-up.

### 3.3. Kaplan–Meier Estimates of Survival Without a Recorded Depression Diagnosis

Figure 2 presents Kaplan–Meier estimates of remaining free from a recorded depression diagnosis, stratified by eating disorder status (A), sex (B), hypertension status (C), and age group (D). Group definitions are provided in the panel legends. Unadjusted differences in time-to-event distributions were assessed using log-rank tests, with results reported in Table 2.

### 3.4. Kaplan–Meier Estimates of Survival Without a Recorded Bipolar Disorder Diagnosis

The Kaplan–Meier survival estimates (Figure 3) demonstrate differential bipolar disorder-free survival trajectories across the examined covariates. In panel A, the survival curves for males and females show only minimal separation over time, indicating limited sex-related differences in bipolar disorder-free survival. Similarly, panel B shows highly comparable survival probabilities among individuals with and without hypertension, suggesting only modest differences between the two groups. In panel C, age groups show more visible separation, with older age categories exhibiting lower bipolar disorder-free survival over follow-up. The most pronounced difference is observed in panel D, where individuals with eating disorders show substantially lower bipolar disorder-free survival compared with those without eating disorders, indicating an elevated long-term risk of a subsequently recorded bipolar disorder diagnosis.

### 3.5. Multivariable Cox Proportional Hazards Regression

In the Cox regression analysis (Table 3), recorded eating disorders were associated with higher adjusted hazards of subsequently recorded depression and bipolar disorder. Patients with a recorded eating disorder had a more than fourfold higher adjusted hazard of depression (aHR = 4.23; 95% CI: 2.11–8.46; *p* < 0.001) and a more than fivefold higher adjusted hazard of bipolar disorder (aHR = 5.45; 95% CI: 2.59–11.44; *p* < 0.001). Age was significantly associated with both outcomes, with adult age groups showing higher hazards compared with the 0–17-year reference group. The highest adjusted hazard was observed in the 45–64-year group for both depression (aHR = 5.53; *p* < 0.001) and bipolar disorder (aHR = 3.72; *p* < 0.001). Female sex was associated with increased depression hazard (aHR = 1.32; *p* < 0.001), but slightly lower bipolar disorder hazard (aHR = 0.92; *p* = 0.019). Hypertension showed a small increase in depression hazard (aHR = 1.09; *p* < 0.001), while it was slightly inversely associated with bipolar disorder (aHR = 0.91; *p* = 0.032). Assessment of Schoenfeld residuals revealed no evidence of violation of the proportional hazards assumption for the ED exposure or the other included covariates.

## 4. Discussion

This large retrospective cohort study found that recorded eating disorders were associated with a more than fourfold higher hazard of subsequently recorded depression and a more than fivefold higher hazard of subsequently recorded bipolar disorder. These findings suggest that recorded eating disorders may serve as markers of subsequent mood disorder diagnoses in routine clinical practice.

The observed sex differences in risk for depression are in line with the epidemiological evidence that depression is approximately 1.5–2 times more common in women than men [15]. A meta-analysis by Rachel H. Salk et al. based on data from multiple countries and age groups revealed a strong and enduring female-to-male ratio in depression prevalence across the lifespan [15]. In our overall depression cohort, female sex was associated with a higher adjusted hazard of a subsequently recorded depression diagnosis than male sex. These differences are likely to be due to a combination of biological factors, psychosocial stressors and sex-related differences in symptom expression and help-seeking behaviour.

Epidemiological studies have generally reported a more equal distribution of sexes in bipolar disorder compared with depression, but sex-specific differences in clinical presentation and course have been consistently reported [16]. A review by Arianna Diflorio and Ian Jones highlighted that while the prevalence of bipolar disorder is broadly similar in men and women, there are important differences in the course of the illness, patterns of symptoms and comorbidities [16]. In our overall bipolar disorder cohort, female sex was associated with a slightly lower adjusted hazard of a subsequently recorded bipolar disorder diagnosis than male sex. These sex associations describe the overall outcome-specific cohorts and do not establish whether the association between eating disorders and subsequently recorded mood disorders differs by sex, as no sex × ED interaction was estimated [16].

Epidemiological studies indicate that depression and bipolar disorder often have an early onset, particularly during adolescence and early adulthood [17,18,19]. Previous longitudinal research has also shown that body-image and eating-related disturbances may prospectively predict increases in depressive symptoms during adolescence [20]. In our study, the adjusted hazard of both depression (aHR = 3.03) and bipolar disorder (aHR = 3.61) was higher in the 18–44-year age group than in the 0–17-year reference group. However, the highest adjusted point estimates for both outcomes were observed in the 45–64-year group (depression: aHR = 5.53; bipolar disorder: aHR = 3.72). These comparisons were made against the 0–17-year reference category and do not demonstrate significant differences between the adult age groups themselves. Moreover, the age coefficients apply to the full cohort and do not indicate that the association between eating disorder status and subsequent mood disorder diagnoses is stronger at a particular age. Therefore, although previous literature supports an early onset of mood disorders and a longitudinal relationship between eating-related pathology and depressive symptoms, our findings should not be interpreted as direct evidence of greater vulnerability in younger individuals, as our analysis examined subsequently recorded diagnoses rather than true age at disease onset.

Hypertension was included as a limited indicator of recorded somatic disease burden [13]. Previous evidence indicates a clinically relevant association between depression and hypertension. A meta-analysis by Meng et al. found that depression was associated with an increased risk of subsequent hypertension [21]. Cardiovascular risk is also relevant across mood disorders. Fiedorowicz reviewed evidence linking mood disorders with cardiovascular disease and noted that elevated vascular risk extends beyond unipolar depression and may also be pronounced in bipolar disorder [22]. From a methodological perspective, differences in healthcare contact and diagnostic recording may influence ascertainment in routine electronic health record data [13]. Adjustment for hypertension addressed this recorded comorbidity but should not be interpreted as controlling for differences in healthcare utilization; residual confounding and surveillance bias remain possible.

The link between eating disorders and later depression is in line with previous longitudinal evidence. The systematic review and meta-analysis by Puccio et al. suggested that eating pathology was an important risk factor for later depression, supporting a temporal relationship and not just co-occurrence cross-sectionally [11].

Previous evidence for bipolar disorder has largely been on comorbidity rather than incident risk, but the literature also supports a meaningful association. Reviews by McElroy and colleagues have concluded that bipolar disorder and eating disorders co-occur at elevated rates, particularly for bulimia nervosa and binge-eating disorder, and that this overlap is associated with greater clinical complexity [23].

More generally, previous clinical studies have demonstrated a substantial psychiatric comorbidity burden among individuals with eating disorders [24]. In a Swedish clinical database study by Ulfvebrand et al., 71% of adults with an eating disorder had at least one additional DSM-IV Axis I disorder, emphasizing the high psychiatric comorbidity in this population [25]. In this regard, our findings add to the literature by examining subsequently recorded depression and bipolar disorder following baseline eating disorder diagnoses. This temporal ordering is consistent with the interpretation that recorded eating disorders may serve as clinical markers of an elevated likelihood of subsequently recorded mood disorders. These associations suggest that assessment and monitoring of mood symptoms may be relevant considerations in the care of patients with eating disorders; however, whether these measures prevent subsequent mood disorders requires direct evaluation in future studies.

Strengths of this study include the large clinical database, longitudinal follow-up, and use of physician-recorded ICD-10 diagnoses from routine clinical care. The longitudinal structure allowed entry-year ED diagnoses to be distinguished from mood disorder diagnoses recorded in subsequent years. The time-to-event approach incorporated differences in observed follow-up duration and enabled estimation of associations after adjustment for the available covariates.

### Limitations

Despite the large overall cohort, few patients had recorded EDs, and subsequent mood disorder events among these patients were sparse. The resulting confidence intervals were wide (depression: 2.11–8.46; bipolar disorder: 2.59–11.44), indicating substantial uncertainty in association magnitudes despite excluding unity. Sparse exposed data also constrained adjustment and caused attempted subgroup models to fail to converge, preventing reliable age- and ED subtype-specific estimation.

The very low proportion of recorded ED diagnoses suggests substantial under-ascertainment. In addition, atypical or less readily classified eating-related behaviors may be under-recognized or not captured by conventional eating disorder coding, as illustrated by emerging evidence on intentional insulin omission and related eating-disorder psychopathology [26]. Coding may preferentially capture severe or clinically complex presentations, while greater psychiatric surveillance could increase detection of mood disorders, potentially inflating the hazard ratios. Unrecorded EDs among comparison patients introduce additional exposure misclassification. Without diagnostic validation, severity measures or direct healthcare utilization measures, the net direction and magnitude of these biases remain uncertain.

A major limitation was restricted confounding control. Psychiatric comorbidity and trauma-history data, including anxiety, substance use and personality disorders, were unavailable for adjustment. BMI, medication exposure, detailed nutritional and metabolic measurements, and socioeconomic characteristics were also unavailable. Adjustment for thyroid and autoimmune disorders was limited; an exploratory autoimmune adjustment was attempted, but diagnoses were sparse and none were recorded among ED-exposed patients. Hypertension was only a limited indicator of recorded somatic morbidity, not a validated measure of healthcare utilization or a substitute for psychiatric covariates. Substantial residual confounding therefore remains possible.

Entry-year exclusions and calendar-year resolution could not establish exact diagnostic intervals or exclude previously unrecognized mood disorders; later diagnoses, particularly bipolar disorder, may therefore reflect delayed recognition rather than new onset. Baseline psychiatric screening information and individual medical charts were unavailable. Outcomes therefore represent newly recorded diagnoses rather than confirmed first onset. Shared vulnerability, pre-existing symptoms and differences in ascertainment may partly explain the associations.

The broad age range and pooled ED subtypes introduce clinical heterogeneity. Hypertension differs in prevalence and relevance across age groups, and depression codes did not distinguish presentations associated with neurocognitive or cerebrovascular disease in older adults. Mortality data were unavailable, preventing assessment of death as a competing event, particularly among older adults and over prolonged follow-up. The reported failure probabilities therefore do not account for competing mortality, and the potential impact of mortality-related loss of observation on the estimates could not be assessed.

Overall age coefficients do not establish age-specific ED effects. Reliance on diagnostic coding at a single clinical centre further limits diagnostic validity and generalizability.

Collectively, these limitations preclude causal interpretation and prevent conclusions about whether screening or early intervention would avert mood disorders or psychiatric deterioration.

## 5. Conclusions

Recorded eating disorders were associated with higher hazards of subsequently recorded depression and bipolar disorder after adjustment for age, sex and hypertension. However, the observational design, limited confounding control, small exposed event counts and potential for unrecognized pre-existing mood disorders preclude causal conclusions. The findings should therefore be interpreted as associations with subsequent diagnostic recording rather than evidence that eating disorders independently cause new-onset mood disorders.

These associations highlight the potential relevance of monitoring mood symptoms in patients with eating disorders. Nevertheless, this study did not evaluate whether screening, early intervention or multidisciplinary care prevents mood disorders or reduces psychiatric deterioration. Such benefits remain possibilities requiring prospective investigation. Larger studies with more comprehensive clinical information are needed to clarify the observed associations and inform targeted approaches to assessment and care.

## Figures and Tables

**Table 1:** Baseline characteristics of the outcome-specific cohorts before follow-up and model-covariate exclusions.

Variable	Category	Depression (*n*, %)	Bipolar (*n*, %)
age	0–17	50,756 (15.71%)	50,913 (15.38%)
	18–44	112,033 (34.67%)	113,378 (34.25%)
	45–64	86,813 (26.87%)	90,917 (27.46%)
	65+	73,504 (22.75%)	75,840 (22.91%)
sex	male	140,573 (43.51%)	142,703 (43.1%)
	female	182,546 (56.49%)	188,358 (56.9%)
recorded eating disorder diagnosis	no	322,980 (99.96%)	330,890 (99.95%)
	yes	140 (0.04%)	172 (0.05%)
hypertension	no	250,020 (77.38%)	253,173 (76.47%)
	yes	73,100 (22.64%)	77,889 (23.53%)

Values are presented as number (percentage). Percentages may not total 100% due to rounding.

**Table 2:** Log-rank comparisons of time to recorded depression and bipolar disorder.

Variable	Depression	*p*-Value	Bipolar	*p*-Value
age	1190.28	*p* < 0.001	233.74	*p* < 0.001
sex	101.45	*p* < 0.001	2.99	*p* = 0.084
recorded eating disorder diagnosis	11.26	*p* < 0.001	20.61	*p* < 0.001
hypertension	263.68	*p* < 0.001	0	*p* = 0.962

Values represent log-rank test statistics. A *p*-value < 0.05 was considered statistically significant.

**Table 3:** Adjusted hazard ratios for subsequently recorded depression and bipolar disorder.

Variable	Category	Depression (aHR)	*p*-Value	Bipolar (aHR)	*p*-Value
age	0–17	ref.		ref.	
	18–44	3.03 [2.63–3.48]	*p* < 0.001	3.61 [3.01–4.33]	*p* < 0.001
	45–64	5.53 [4.81–6.36]	*p* < 0.001	3.72 [3.09–4.46]	*p* < 0.001
	65+	5.42 [4.70–6.25]	*p* < 0.001	3.12 [2.57–3.78]	*p* < 0.001
sex	male	ref.		ref.	
	female	1.32 [1.26–1.38]	*p* < 0.001	0.92 [0.86–0.98]	*p* = 0.019
recorded eating disorder diagnosis	no	ref.		ref.	
	yes	4.23 [2.11–8.46]	*p* < 0.001	5.45 [2.59–11.44]	*p* < 0.001
hypertension	no	ref.		ref.	
	yes	1.09 [1.04–1.15]	*p* < 0.001	0.91 [0.84–0.99]	*p* = 0.032

Values are adjusted hazard ratios (aHRs) with 95% confidence intervals (CIs). Separate Cox proportional hazards models were fitted for each outcome, with baseline eating disorder status, age group (0–17, 18–44, 45–64 and ≥65 years), sex, and hypertension status entered simultaneously. Each estimate is therefore adjusted for the other variables in the model. ref., reference category.

## Data Availability

The data that support the findings of this study are available from Clinical Centre of the University of Debrecen, Hungary but restrictions apply to the availability of these data, which were used under license for the current study, and so are not publicly available. Data are however available from the authors upon reasonable request and with permission of Clinical Centre of the University of Debrecen, Hungary.

## Supplementary Materials

The following supporting information can be downloaded at: https://www.mdpi.com/article/10.3390/nu18203404/s1, Figure S1. Participant selection and derivation of the outcome-specific Cox analysis samples. ED, eating disorder recorded in the entry year. The depression and bipolar disorder cohorts overlap and are not mutually exclusive. Table S1. STROBE Statement; Table S2. Participant counts, out-come events, person-time, and crude rates of first recorded mood disorder diagnoses by base-line eating disorder status; Table S3. Baseline eating disorder codes and subsequent recorded mood disorder events in the Cox analysis samples.
