# Supplementary material for: Association Between Eating Disorders and Subsequently Recorded Depression and Bipolar Disorder: A Retrospective Cohort Study
Source: Nutrients. 2026 Oct 17;18(20):3404. doi: 10.3390/nu18203404 (PMC13708549; doi:10.3390/nu18203404)
Supplement: Supplementary file 1 [file nutrients-18-03404-s001.zip › STROBE.pdf]

Table S1. STROBE Statement—Checklist of items that should be included in reports of ***cohort studies***

Manuscript: Association Between Eating Disorders and the Subsequent Incidence of Depression and Bipolar Disorder: A Retrospective Cohort Study

	Item No	Recommendation	Response and location in manuscript
**Title and abstract**	1	(*a*) Indicate the study's design with a commonly used term in the title or the abstract	**Title and Abstract, pp. 1–2.** The study is identified as a retrospective cohort study in both the title and the Abstract.
		(*b*) Provide in the abstract an informative and balanced summary of what was done and what was found	**Abstract, p. 2.** The Abstract summarizes the clinical setting, 2007–2022 observation period, routinely recorded diagnoses, outcome-specific entry-year exclusions, survival analysis and principal associations. ED-associated HRs are 4.23 (95% CI 2.11–8.46) for depression and 5.45 (2.59–11.44) for bipolar disorder.
**Introduction**			
Background/rationale	2	Explain the scientific background and rationale for the investigation being reported	**Introduction, pp. 2–3.** The rationale addresses the clinical burden and comorbidity of eating and mood disorders, shared vulnerability, limitations of cross-sectional evidence, and the need to investigate the longitudinal ordering of recorded diagnoses.
Objectives	3	State specific objectives, including any prespecified hypotheses	**Introduction, final paragraph, p. 3.** The objective was to examine whether recorded eating disorders were associated with subsequently recorded depression and bipolar disorder. The stated hypothesis was that patients with EDs would have higher subsequent hazards than patients without recorded EDs.
**Methods**			
Study design	4	Present key elements of study design early in the paper	**§2.1, p. 3; §2.2, p. 4.** This retrospective longitudinal cohort used routinely collected hospital records. Separate outcome-specific cohorts were analysed, with fixed entry-year ED exposure and follow-up to the first subsequent recorded outcome or last available observation.
Setting	5	Describe the setting, locations, and relevant dates, including periods of recruitment, exposure, follow-up, and data collection	**§§2.1–2.2, pp. 3–4.** The setting was the Clinical Center of the University of Debrecen, Hungary. Records covered 2007–2022. The index year was each patient's first recorded calendar year; ED exposure was assessed in that year, and follow-up was measured in calendar-year units.

	Item No	Recommendation	Response and location in manuscript
Participants	6	(*a*) Give the eligibility criteria, and the sources and methods of selection of participants. Describe methods of follow-up	**§2.2, p. 4; §3.1, p. 5.** The manuscript specifies hospital patients with at least two visits and complete clinical records. Patients with the relevant mood disorder recorded in their entry year were excluded from the corresponding cohort. No fixed pre-entry washout was required. Cox analyses used patients with positive follow-up and complete model covariates. Later ED diagnoses did not alter baseline exposure classification.
		(*b*) For matched studies, give matching criteria and number of exposed and unexposed	**§§2.1–2.2, pp. 3–4.** Not applicable: the exposed and unexposed cohorts were not matched.
Variables	7	Clearly define all outcomes, exposures, predictors, potential confounders, and effect modifiers. Give diagnostic criteria, if applicable	**§§2.2–2.3, p. 4; Table 3, p. 8.** Baseline ED exposure used ICD-10 codes F50.10, F50.20, F50.30, F50.40 and F50.50. Outcomes were first recorded depression (F32/F33) or bipolar disorder (F31) in subsequent calendar years. Model covariates were age category, sex and recorded hypertension. The comparison group had no qualifying entry-year ED diagnosis.
Data sources/ measurement	8*	For each variable of interest, give sources of data and details of methods of assessment (measurement). Describe comparability of assessment methods if there is more than one group	**§§2.1–2.3, pp. 3–4; Limitations, pp. 10–11.** Both exposure groups were identified in the same secondary hospital dataset using physician-recorded ICD-10 diagnoses and demographic and temporal fields. Medical charts and standardized psychiatric screening information were unavailable for independent validation. BMI, medication and detailed nutritional/metabolic measurements were unavailable. Differences in diagnostic ascertainment are acknowledged.
Bias	9	Describe any efforts to address potential sources of bias	**§§2.2–2.4, pp. 4–5; Limitations, pp. 10–11.** The same index-year definition was applied to both groups; future ED diagnoses were not used to assign baseline exposure. Entry-year outcome diagnoses were excluded, and Cox models adjusted for available covariates. Under-ascertainment, surveillance bias, residual confounding, delayed recognition, exposure misclassification and unobserved competing mortality are discussed.
Study size	10	Explain how the study size was arrived at	**§2.1, p. 3; §3.1, p. 5.** Study size arose from the available 2007–2022 hospital database and outcome-specific eligibility and analysis restrictions. The source contained 333,807 unique patients and 805,989 records. Initial depression and bipolar cohorts included 323,120 and 331,062 patients; final Cox samples included 163,199 and 169,342, respectively.

	Item No	Recommendation	Response and location in manuscript
Quantitative variables	11	Explain how quantitative variables were handled in the analyses. If applicable, describe which groupings were chosen and why	**§§2.2–2.3, p. 4; Tables 1 and 3; response to Reviewer 2, comment 5.** Age was grouped as 0–17, 18–44, 45–64 and ≥65 years, with 0–17 as reference, representing pragmatic life-stage categories. An initial continuous-age specification showed non-proportionality; no violation was detected in the final categorical models. Follow-up was measured in calendar-year units; sex, ED and hypertension were binary variables.
Statistical methods	12	(*a*) Describe all statistical methods, including those used to control for confounding	**§2.4, pp. 4–5; §§3.5–3.7, pp. 7–8; verified Cox output.** Stata 18 was used for Cox regression with Breslow handling of ties, simultaneously including ED, age category, sex and hypertension. Covariate choice reflected clinical relevance and data availability. HRs and 95% CIs were reported; proportional hazards were assessed with Schoenfeld residuals. Kaplan–Meier curves and log-rank comparisons described survival differences; model-based S(t) and 1 − S(t) were distinguished from competing-risk incidence.
		(*b*) Describe any methods used to examine subgroups and interactions	**§2.4, p. 5; Limitations, p. 10.** Exploratory Cox models within age groups and ED subtypes were attempted. Sparse exposed observations and events led to non-convergence, preventing reliable subgroup estimation. Estimates from non-convergent models were not interpreted. No formal interaction estimates are presented.
		(*c*) Explain how missing data were addressed	**§3.1, p. 5; verified Cox estimation samples.** Cox models used complete cases for the included model covariates among patients with positive follow-up. Three additional patients were excluded from each outcome-specific survival sample during model estimation, leaving 163,199 depression and 169,342 bipolar participants. Variables unavailable in the secondary dataset could not be included in adjustment.
		(*d*) If applicable, explain how loss to follow-up was addressed	**§2.2, p. 4; Limitations, p. 11.** Follow-up ended at the first recorded outcome or last available observation; patients without a recorded outcome were censored at that last observation. Vital status and death dates were unavailable. Potential effects of mortality-related loss of observation and unmeasured competing death are acknowledged.

	Item No	Recommendation	Response and location in manuscript
		(*e*) Describe any sensitivity analyses	**Limitations, pp. 10–11; response to Reviewer 1, Methodology comment.** Additional adjustment for autoimmune disorders was explored as a sensitivity analysis. Recorded autoimmune diagnoses were sparse, with none among ED-exposed patients, limiting this analysis. No reliable sensitivity estimate or claim of robustness from this attempted adjustment is presented.
**Results**			
Participants	13*	(a) Report numbers of individuals at each stage of study—eg numbers potentially eligible, examined for eligibility, confirmed eligible, included in the study, completing follow-up, and analysed	**§§2.1 and 3.1, pp. 3 and 5; Supplementary Table S1; verified Stata output.** Source: 333,807 patients. After entry-year outcome exclusions, the depression cohort contained 323,120 patients (140 ED; 322,980 unexposed), and the bipolar cohort 331,062 (172 ED; 330,890 unexposed). Positive-follow-up samples were 163,202 and 169,345. Final Cox samples were 163,199 (68 ED; 163,131 unexposed) and 169,342 (82 ED; 169,260 unexposed), respectively.
		(b) Give reasons for non-participation at each stage	**§§2.2 and 3.1, pp. 4–5; verified Stata output.** Entry-year outcome exclusions removed 10,687 patients from the depression cohort and 2,745 from the bipolar cohort. Nonpositive follow-up excluded another 159,918 depression patients (72 ED; 159,846 unexposed) and 161,717 bipolar patients (90 ED; 161,627 unexposed). Model-covariate exclusions removed three further unexposed patients per outcome. Counts refer to patients, not hospital-record rows.
		(c) Consider use of a flow diagram	**§§2.1–2.2 and 3.1, pp. 3–5; Supplementary Table S1.** Participant selection is described in the Methods and Results; initial cohorts appear in Table 1 and final model samples in Supplementary Table S1. The stages and exclusion counts are detailed in checklist items 13(a) and 13(b). A separate flow diagram is not included.
Descriptive data	14*	(a) Give characteristics of study participants (eg demographic, clinical, social) and information on exposures and potential confounders	**§3.1 and Table 1, p. 5; Supplementary Table S1; Limitations, pp. 10–11.** Table 1 describes age, sex, hypertension and recorded ED frequency in each outcome-specific cohort. Supplementary Table S1 gives final-model participant counts, events and person-time separately by ED status. Unavailable psychiatric, treatment, BMI, metabolic and socioeconomic information is identified in the Limitations.

	Item No	Recommendation	Response and location in manuscript
		(b) Indicate number of participants with missing data for each variable of interest	**Table 1 and §3.1, p. 5; verified Cox output.** Table 1 gives age-category counts for 323,106 depression-cohort and 331,048 bipolar-cohort patients, and sex counts for 323,119 and 331,061, respectively. The final models used complete covariate data, excluding three patients per outcome after survival eligibility. Per-variable missingness is not separately tabulated.
		(c) Summarise follow-up time (eg, average and total amount)	**Supplementary Table S1; verified survival output.** Depression follow-up totalled 939,013 person-years: 322 among ED-exposed and 938,691 among unexposed patients. Bipolar follow-up totalled 1,002,457 person-years: 416 exposed and 1,002,041 unexposed. Person-time is rounded to whole years; observed follow-up extended to 15 years.
Outcome data	15*	Report numbers of outcome events or summary measures over time	**§3.1, p. 5; Supplementary Table S1.** The final depression model included 7,489 events: 8 among 68 ED-exposed and 7,481 among 163,131 unexposed patients. The bipolar model included 3,552 events: 7 among 82 exposed and 3,545 among 169,260 unexposed patients. The supplement reports corresponding crude rates per 1,000 person-years with 95% CIs.
Main results	16	(*a*) Give unadjusted estimates and, if applicable, confounder-adjusted estimates and their precision (eg, 95% confidence interval). Make clear which confounders were adjusted for and why they were included	**§§3.5–3.6 and Tables 2–3, pp. 7–8; Supplementary Table S1.** Crude ED/unexposed rates per 1,000 person-years were 24.845/7.970 for depression and 16.827/3.538 for bipolar disorder, with 95% CIs in Table S1; unadjusted survival comparisons used log-rank tests. ED aHRs were 4.23 (2.11–8.46) and 5.45 (2.59–11.44), respectively, adjusted for age, sex and hypertension. Selection reflected demographic relevance and available somatic morbidity data; substantial residual confounding remains possible.
		(*b*) Report category boundaries when continuous variables were categorized	**§2.3, p. 4; Tables 1 and 3, pp. 5 and 8.** Age-category boundaries were 0–17, 18–44, 45–64 and ≥65 years; 0–17 years was the reference category.
		(*c*) If relevant, consider translating estimates of relative risk into absolute risk for a meaningful time period	**§2.4, pp. 4–5; Figures 1 and 4, pp. 6 and 8; Supplementary Table S1.** The analysis focuses on associations. Model-based survival and failure probabilities and crude diagnosis rates provide complementary information; HRs were not directly translated into fixed-horizon absolute risks. Mortality data were unavailable, and the failure curves are explicitly not competing-risk cumulative incidence estimates.

	Item No	Recommendation	Response and location in manuscript
Other analyses	17	Report other analyses done—eg analyses of subgroups and interactions, and sensitivity analyses	**§2.4, p. 5; Limitations, pp. 10–11.** Attempts to estimate age- and ED-subtype-specific associations did not converge because of sparse exposed observations/events. Exploratory autoimmune adjustment was limited by sparse diagnoses. These constraints are disclosed without interpreting unstable estimates. ROC/AUC analyses were removed to retain the study's associational focus.
**Discussion**			
Key results	18	Summarise key results with reference to study objectives	**Discussion, pp. 9–10; Conclusions, pp. 11–12.** Patients with entry-year recorded EDs had higher hazards of subsequently recorded depression and bipolar disorder after adjustment for age, sex and hypertension. The conclusions relate these associations to the longitudinal objective while distinguishing recorded diagnoses from confirmed first onset and causal effects.
Limitations	19	Discuss limitations of the study, taking into account sources of potential bias or imprecision. Discuss both direction and magnitude of any potential bias	**Limitations, pp. 10–11.** Limitations include sparse ED events and wide CIs, ED under-recording, diagnostic misclassification, surveillance bias, residual confounding, unavailable clinical and treatment variables, delayed diagnosis, annual temporal resolution, pooled ED subtypes, competing mortality and single-centre sampling. Surveillance could inflate associations; exposure misclassification adds uncertainty. The net direction and magnitude of these biases could not be determined.
Interpretation	20	Give a cautious overall interpretation of results considering objectives, limitations, multiplicity of analyses, results from similar studies, and other relevant evidence	**Discussion and Limitations, pp. 9–11; Conclusions, pp. 11–12.** The associations are considered alongside previous longitudinal and comorbidity research, shared vulnerability and substantial uncertainty. The Limitations and Conclusions explicitly preclude causal and preventive conclusions. Monitoring is framed as a clinical consideration; whether screening or early intervention prevents mood disorders requires prospective evaluation. Overall age coefficients do not establish age-specific ED effects.
Generalisability	21	Discuss the generalisability (external validity) of the study results	**Limitations, pp. 10–11.** Generalisability is limited by the single clinical centre, reliance on coded diagnoses and likely ED under-ascertainment. Recorded EDs may preferentially represent severe or clinically complex presentations. Broad age groups and pooled ED subtypes introduce heterogeneity; results should not be generalized directly to all individuals with EDs or to other healthcare settings.

	Item No	Recommendation	Response and location in manuscript
**Other information**			
Funding	22	Give the source of funding and the role of the funders for the present study and, if applicable, for the original study on which the present article is based	**Funding, p. 12.** The study received EKÖP-26-3-II scholarship support awarded to Amr Sayed Ghanem and EKÖP-26-2 support awarded to Vanessza Hadar, funded by the Ministry for Science and Technology from the National Research, Development and Innovation Fund. The funding statement does not specify the funders' role in the study.

*Give information separately for exposed and unexposed groups.

Note: An Explanation and Elaboration article discusses each checklist item and gives methodological background and published examples of transparent reporting. The STROBE checklist is best used in conjunction with this article (freely available on the Web sites of PLoS Medicine at http://www.plosmedicine.org/, Annals of Internal Medicine at http://www.annals.org/, and Epidemiology at http://www.epidem.com/). Information on the STROBE Initiative is available at http://www.strobe-statement.org.
