# Supplementary material for: Association Between Eating Disorders and Subsequently Recorded Depression and Bipolar Disorder: A Retrospective Cohort Study
Source: Nutrients. 2026 Oct 17;18(20):3404. doi: 10.3390/nu18203404 (PMC13708549; doi:10.3390/nu18203404)

Supplementary Table S2. Participant counts, outcome events, person-time, and crude rates of first recorded mood disorder diagnoses by baseline eating disorder status.

**Outcome**	**Baseline ED status**	**Participants**	**Events**	**Person-years**	**Rate per 1,000 person-years (95% CI)**
Depression	No recorded ED	163,131	7,481	938,691	7.970 (7.791–8.152)
Depression	Recorded ED	68	8	322	24.845 (12.425–49.680)
Bipolar disorder	No recorded ED	169,260	3,545	1,002,041	3.538 (3.423–3.656)
Bipolar disorder	Recorded ED	82	7	416	16.827 (8.022–35.296)

ED, eating disorder; CI, confidence interval. Results refer to the respective Cox estimation samples. Rates are unadjusted and concern first recorded diagnoses during follow-up. Person-time is rounded to whole person-years.

The bipolar unexposed person-time is calculated from the reported total: 1,002,457 − 416 = 1,002,041 person-years.

Supplementary Table S3. Baseline eating disorder codes and subsequent recorded mood disorder events in the Cox analysis samples.

**Baseline ICD-10 code**	**Depression analysis: patients**	**Depression events**	**Bipolar analysis: patients**	**Bipolar events**
F50.0	31	4	43	4
F50.1	3	1	3	0
F50.2	12	2	10	2
F50.3	2	1	2	1
F50.4	1	0	2	0
F50.5	3	0	3	0
F50.8	14	0	16	0
F50.9	6	0	7	0
**Any qualifying ED code: unique patients**	**68**	**8**	**82**	**7**

Counts refer to the final Cox estimation samples: 163,199 patients for depression and 169,342 for bipolar disorder. ED codes were identified in the entry year. Patients could have multiple baseline ED codes, so code-specific patient counts are not mutually exclusive. The

total row counts each patient and outcome event once. Events represent first recorded diagnoses during follow-up.

Supplementary Figure S1. Participant selection and derivation of the outcome-specific Cox analysis samples. ED, eating disorder recorded in the entry year. The depression and bipolar disorder cohorts overlap and are not mutually exclusive.

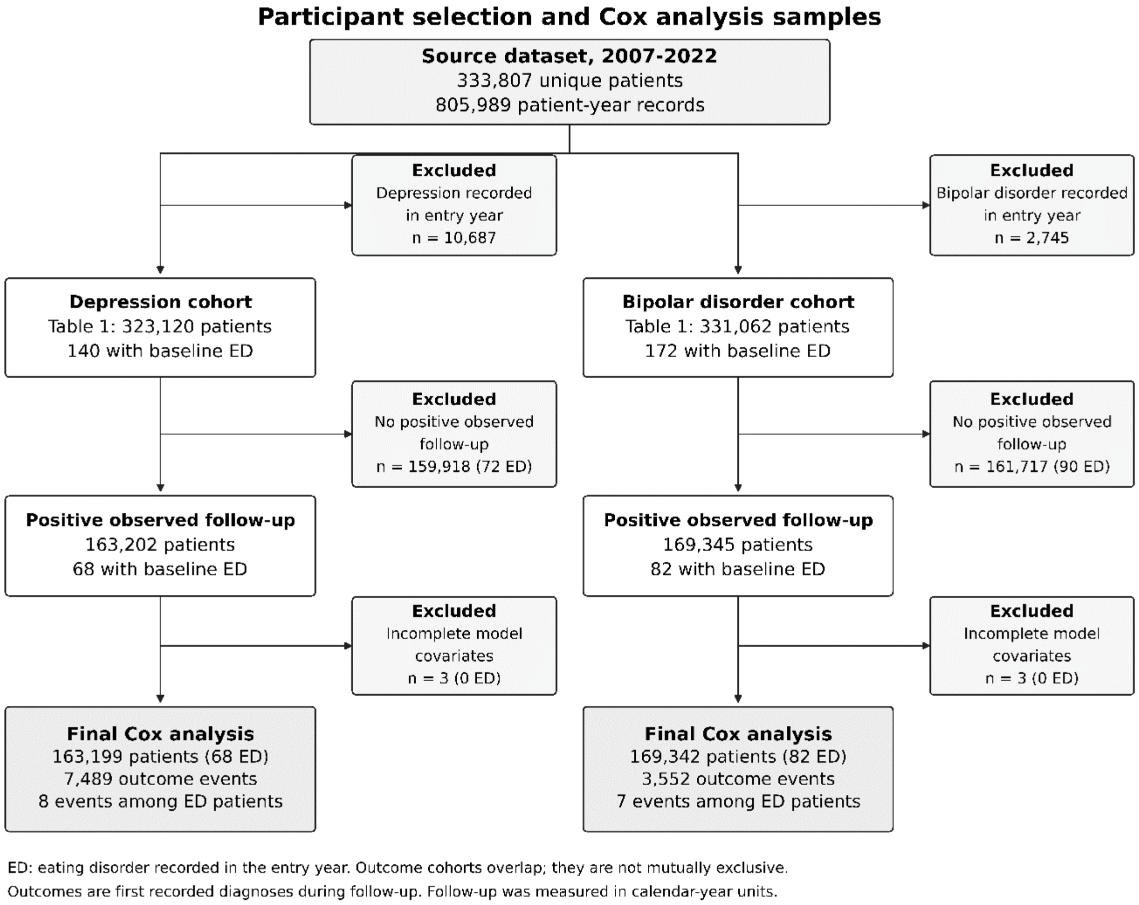

Supplement: Supplementary file 1 [file nutrients-18-03404-s001.zip › supplementary material.pdf]
